# The Impact of Two Different Transfusion Strategies on Patient Immune Response during Major Abdominal Surgery: A Preliminary Report

**DOI:** 10.1155/2014/945829

**Published:** 2014-04-03

**Authors:** Kassiani Theodoraki, Maria Markatou, Demetrios Rizos, Argyro Fassoulaki

**Affiliations:** ^1^Department of Anesthesiology, Aretaieion University Hospital, 11528 Athens, Greece; ^2^Hormonal and Biochemical Laboratory, Aretaieion University Hospital, 11528 Athens, Greece

## Abstract

Blood transfusion is associated with well-known risks. We investigated the difference between a restrictive versus a liberal transfusion strategy on the immune response, as expressed by the production of inflammatory mediators, in patients subjected to major abdominal surgery procedures. Fifty-eight patients undergoing major abdominal surgery were randomized preoperatively to either a restrictive transfusion protocol or a liberal transfusion protocol (with transfusion if hemoglobin dropped below 7.7 g dL^−1^ or 9.9 g dL^−1^, respectively). In a subgroup of 20 patients randomly selected from the original allocation groups, blood was sampled for measurement of IL-6, IL-10, and TNF**α**. Postoperative levels of IL-10 were higher in the liberal transfusion group on the first postoperative day (49.82 ± 29.07 vs. 15.83 ± 13.22 pg mL^−1^, *P* < 0.05). Peak postoperative IL-10 levels correlated with the units of blood transfused as well as the mean duration of storage and the storage time of the oldest unit transfused (*r* ^2^ = 0.38, *P* = 0.032, *r* ^2^ = 0.52, *P* = 0.007, and *r* ^2^ = 0.68, *P*<0.001, respectively). IL-10 levels were elevated in patients with a more liberal red blood cell transfusion strategy. The strength of the association between anti-inflammatory IL-10 and transfusion variables indicates that IL-10 may be an important factor in transfusion-associated immunomodulation. This trial is registered under ClinicalTrials.gov Identifier: NCT02020525.

## 1. Introduction

Major abdominal surgery can often be complicated by massive hemorrhage with all the sequelae that profound anemia entails in this setting [1]. Therefore, blood transfusion is considered a cornerstone of perioperative care practice and is used to augment oxygen delivery in the hope of avoiding the deleterious effects of serious anemia and the resulting oxygen debt, especially in vulnerable patients [2].

On the other hand, the administration of blood products is associated with well-described adverse consequences. In particular, allogeneic red cell transfusions can potentially lead to the transmission of viral infections, febrile nonhemolytic transfusion reactions, or alloimmunization to human leukocyte antigens [3]. There is always a small but distinct possibility for bacterial contamination as well as for errors in blood administration. Furthermore, the prolonged storage of blood products may decrease the ability of the red cell to transport or deliver oxygen through an abnormal microcirculation [4–6]. There is also growing concern about limited supply and the escalating cost of blood transfusion.

More importantly, transfusion of blood products has been linked with the induction of clinically important immune suppression, which may unfavorably affect the postoperative course by increasing tumor recurrence rate or the potential for serious nosocomial infections [7, 8]. The surgical trauma itself causes a systemic inflammatory response through activation of various cellular and humoral cascade systems [9]. Whenever blood transfusion is needed, a secondary inflammatory insult might ensue, which enhances the initial inflammatory response evoked by the surgical procedure [10, 11]. The mechanisms involved in the immunomodulatory effect of allogeneic blood transfusion have not been elucidated yet, but it has been suggested that these adverse effects may be mediated by white blood cells present in transfused cellular blood components and the generation of inflammatory mediators [12]. Transfusion-associated immunomodulation (TRIM) has also been associated with the duration of storage of blood components [5, 13, 14] and the impairment in the balance between proinflammatory (tumor necrosis factor (TNF*α*), interleukin-1 (IL-1), IL-6), and anti-inflammatory circulating cytokines (IL-4, IL-10) [15, 16].

For all these reasons, there is a trend leading to reassessment of transfusion strategies and over the recent years there have been recommendations that physicians lower the trigger of hemoglobin (Hb) level at which patients are transfused. However, the level of Hb that most accurately predicts the need for blood transfusion has been widely debated and transfusion practices still remain highly variable and controversial.

We have previously reported the results of the primary and secondary outcomes of a randomized study aiming to investigate the impact of a restrictive transfusion protocol on the magnitude of reduction in blood transfusion in a typically mixed general surgery population subjected to major abdominal surgery [17]. The main finding of that study was a reduction in red blood cell usage with the implementation of a restrictive transfusion regimen. Notably, this was achieved without adversely affecting clinical outcome in the population studied.

The aim of this secondary analysis performed on a subgroup of 20 patients from the original study was to determine whether there are any differences in the postoperative immunologic response, as expressed by the production of inflammatory mediators, between a restrictive approach to red cell transfusion and a more liberal strategy.

## 2. Methods

### 2.1. Study Subjects

The study was approved by the Institutional Review Board of Areteion University Hospital. The study protocol has been presented in detail previously [17]. Patients scheduled for elective upper major abdominal surgery were enrolled in the study after providing written informed consent. Exclusion criteria were a history of bleeding diathesis and refusal of transfusions for religious reasons or a history of active ischemic heart disease (unstable angina or myocardial infarction within 6 months preceding the scheduled operation). Moreover, patients with preexisting infectious or autoimmune diseases were excluded from participation as well as those patients having used corticosteroids or immunosuppressive drugs within 6 months.

Patients were randomized preoperatively to one of two intraoperative and postoperative transfusion strategies. Sealed opaque envelopes containing odd and even numbers were chosen at random for patient assignment. Patients assigned to the liberal strategy were transfused when their hemoglobin concentration fell below 9.9 g dL^−1^, aiming at maintaining hemoglobin at or above 10 g dL^−1^. Patients allocated to the restrictive transfusion strategy were transfused only when their hemoglobin concentration decreased below 7.7 g d dL^−1^ and were then maintained at hemoglobin concentrations between 7.7 and 9.9 g d dL^−1^.

### 2.2. Transfusion Management

All patients were operated under using the same anesthetic protocol, while antibiotic prophylaxis and postoperative analgesia were also standardized. Transfusion guidelines and group assignment were followed both intraoperatively and postoperatively. Both the surgical team and anesthesiologists responsible for the patient were informed as to the allocation group. Intraoperative transfusions were supervised by the anesthesiologist in charge of the protocol and postoperative transfusions by both the surgeon and anesthesiologist in charge. Ward personnel were informed about transfusion strategy assignment to ensure compliance with the protocol with the aim to treat transfusion trigger deviations as protocol violations. Moreover, adherence to the transfusion protocol was ensured by blood transfusion being prescribed only by the research team involved in the study. All transfusions were nonleukodepleted packed red blood cells (RBCs) stored in citrate-phosphate dextrose adenine-1 (CPDA-1). The maximum duration of storage of erythrocyte units is 42 days according to policies followed by blood banks across the world [18]. The date of collection of each unit transfused was retrieved from blood bank records and the length of storage of each unit transfused between the date of collection and the date of transfusion was calculated. Transfusions were administered one unit at a time and hemoglobin concentration was measured in all study patients with the HemoCue 201 DM device (HemoCue, Inc., Cypress, CA, USA) after each red blood cell unit had been transfused. Compliance to the transfusion protocol was monitored by daily measurements of hemoglobin concentration in each patient.

### 2.3. Study Endpoints and Postoperative Follow-Up

Primary outcome measure of the original study was red blood cell usage, as expressed by the number of units transfused per patient as well as the difference in the incidence of blood transfusions between the two randomization groups [17]. In this secondary analysis performed on a subgroup of 20 patients randomly selected from the original allocation groups, blood was sampled for measurement of IL-6, IL-10, and TNF*α* preoperatively, 6 hours, one day, and three days postoperatively. Time of mobilization, time of first liquid and solid food intake, and length of postoperative hospital stay were also recorded for each patient. Additionally, patients were followed up on a daily basis until hospital discharge during which time the incidence of all postoperative infectious complications was recorded. Finally, two variables were used to study the effect of the length of storage of the RBC units transfused to each patient: (1) the mean length of storage of all RBC units transfused and (2) the length of storage of the oldest unit transfused per patient.

### 2.4. Cytokine Analysis

In a subgroup of patients (10 patients randomly selected from each transfusion policy allocation group) IL-6, IL-10, and TNF*α* were measured. Peripheral venous blood was drawn at the following time points: preoperatively, 6 hours, one day, and three days postoperatively. All samples were collected in sterile tubes (Vacutainer, Becton-Dickinson, Heidelberg, Germany) and were immediately centrifuged and the supernatant was stored at −60°C until assay. Quantitative determination of cytokine levels was performed using commercially available sensitive immunoassay kits (Quantikine HS human IL-6, Quantikine HS IL-10, and Quantikine HS human TNF*α* for IL-6, IL-10, and TNF*α*, resp.) (R&D Systems Inc. 614 McKinley Place NE, MN, USA), according to the recommendations of the manufacturer. Detection sensitivity was 0.039 pg mL^−1^ for IL-6, 3.9 pg mL^−1^ for IL-10, and 0.106 pg mL^−1^ for TNF*α*. The coefficient of variability of the method was 6.5–9.6% for IL-6, 4.3–7.5% for IL-10, and 5.3–16.7% for TNF*α*. All assays were performed in duplicate and averaged data were used in the subsequent analysis.

### 2.5. Statistics

Power calculation and estimation of sample size were based on the primary outcome measure of the original trial and have previously been described in detail [17]. Variables were tested for normality of distributions with the Kolmogorov-Smirnov test. Comparisons of numeric data between the two groups were performed with the unpaired *t*-test or the Wilcoxon rank sum test for independent samples, depending on whether the variables followed a normal or nonnormal distribution. The chi-square test or Fisher's exact test, as appropriate, was used for comparisons of categorical data. Correlation between data was tested by using the Pearson product moment correlation coefficient test. Stepwise multiple linear regression analysis was performed in order to adjust for the effect of confounding and to investigate the independent predictive value of variables. The postoperative changes in Hb levels as well as serial changes in IL-6, Il-10, and TNF*α* levels were analyzed with two-factor mixed design analysis of variance with repeated measures for one factor (time). The two factors were the subject group and time and the Student-Newman-Keuls method was used* post hoc* for pairwise multiple comparisons. Results are expressed as mean SD or as median (25th–75th percentiles) depending on normality of distributions. A value of *P* < 0.05 was considered as statistically significant. Statistical analysis was performed by the use of SPSS for Windows v.16.0 statistical software (SPSS Inc., Chicago, Il, USA).

## 3. Results

**Figure 1:**
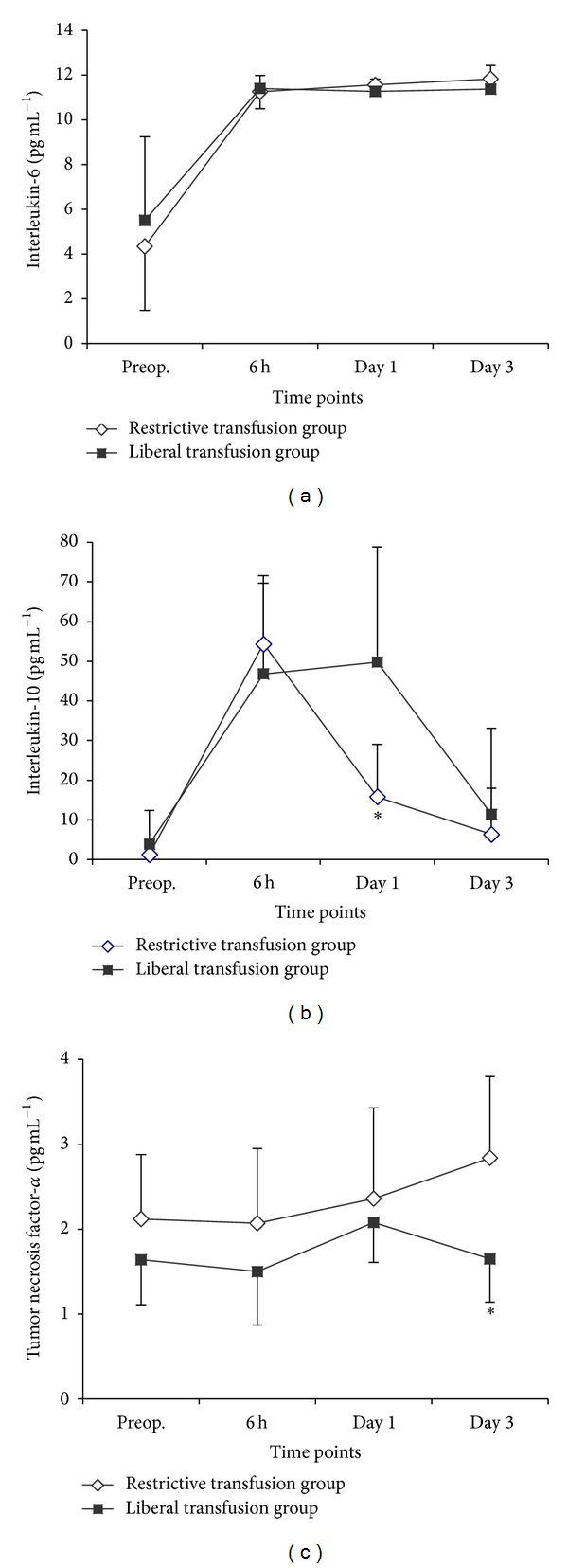
(a) Serial changes in perioperative IL-6 levels. Data are presented as mean ± SD. No intergroup differences were demonstrated. (*P* < 0.001, effect of time; *P* = 0.462, group by time interaction). (b) Serial changes in perioperative IL-10 levels. Data are presented as mean ± SD. Postoperative systemic induction of IL-10 was significantly exaggerated in the liberal transfusion group 24 h postoperatively. (*P < 0.05 for intergroup comparison; *P* < 0.001, effect of time; *P* < 0.001, group by time interaction). (c) Serial changes in perioperative TNF*α* levels. Data are presented as mean ± SD. There was a difference between the two groups on the third postoperative day. (*P < 0.05 for intergroup comparison; *P* = 0.842, effect of time; *P* = 0.029, group by time interaction).

The 20 patients randomly selected from the two transfusion allocation groups did not differ significantly in demographic characteristics, namely, age, weight, height, sex, American Society of Anesthesiologists (ASA) distribution, and the type of surgical procedures performed. The postoperative serial changes in the circulating levels of IL-6, IL-10, and TNF*α* in these two subgroups of patients are summarized in Figure 1. IL-6 was distinctly higher from baseline at all time points in both subgroups. No intergroup differences were demonstrated for IL-6 at any time point. IL-10 also exhibited a postoperative increase as compared to baseline in the two transfusion policy groups, which was obvious 6 and 24 hours postoperatively, with a subsequent decline to near baseline ranges at the end of the observation period. However, postoperative systemic induction of IL-10 was significantly exaggerated in patients subjected to a higher volume of transfusion (*P* < 0.05 for intergroup comparison 24 hours postoperatively). Postoperative concentrations of TNF*α* were not significantly different from baseline in either subgroup. TNF*α* levels were lower on the third postoperative day in the liberal transfusion group as compared to the restrictive group (*P* < 0.05 for intergroup comparison).

**Figure 2:**
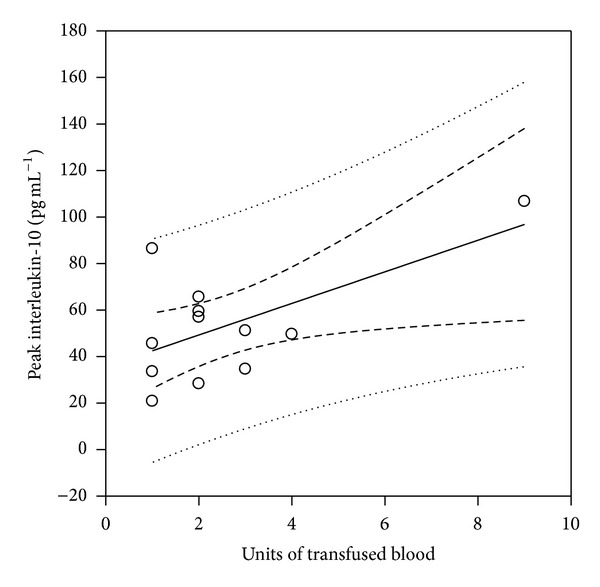
Scatter plot diagram of peak postoperative IL-10 values versus the number of units transfused, depicting a significant correlation (*r* ^2^ = 0.38, *P* = 0.032).

**Figure 3:**
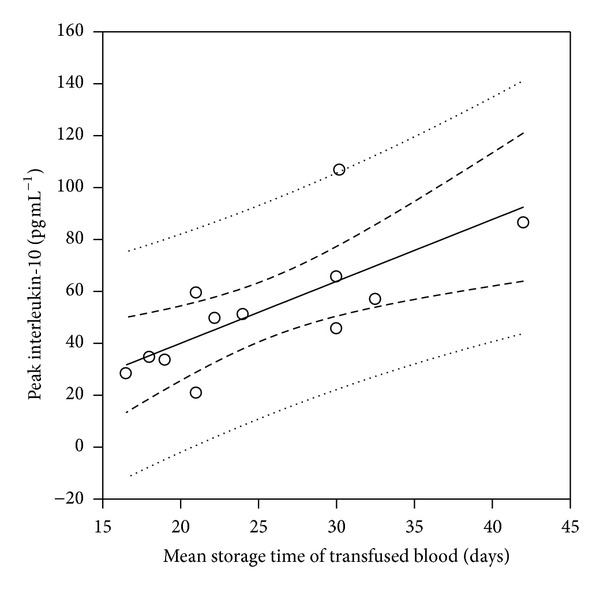
Scatter plot diagram of peak postoperative IL-10 values versus the mean duration of storage of transfused blood (in days). The storage time of transfused blood demonstrated a strong correlation to peak IL-10 values (*r* ^2^ = 0.52, *P* = 0.007).

**Figure 4:**
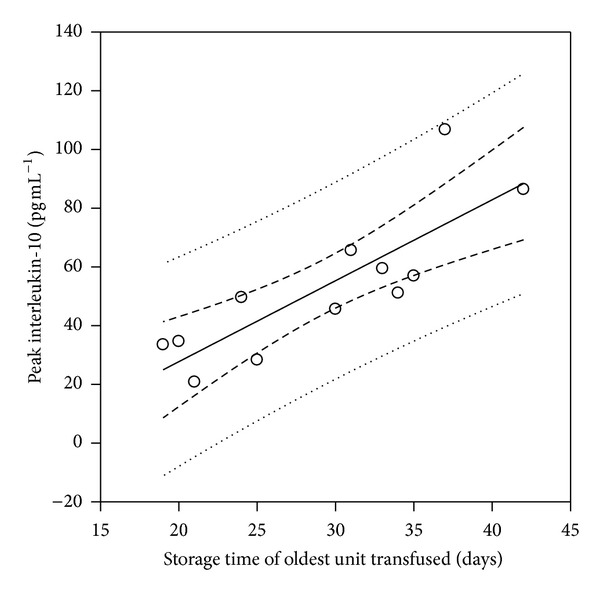
Scatter plot diagram of peak postoperative IL-10 values versus the duration of storage (in days) of the oldest unit of blood transfused. A strong correlation between the storage time of the oldest unit transfused and peak IL-10 values was demonstrated (*r* ^2^ = 0.68, *P* < 0.001).

Peak postoperative IL-10 levels were found to correlate significantly with the units of blood transfused (*r* ^2^ = 0.38, *P* = 0.032) (Figure 2). Strong correlations between peak postoperative IL-10 values and the mean duration of storage of blood transfused (in days) (Figure 3) as well as the storage time (in days) of the oldest unit transfused (Figure 4) were also demonstrated (*r* ^2^ = 0.52, *P* = 0.007 and *r* ^2^ = 0.68, *P* < 0.001, resp.). No correlations for the other two mediators were demonstrated. Furthermore, we entered the units of blood transfused, the mean age of the blood transfused, and the storage time of the oldest unit transfused, which were associated with peak IL-10 values by univariable analysis, in a multivariable stepwise linear regression analysis model. Multivariable regression analysis showed that all three factors were independent variables significantly associated with peak postoperative IL-10 levels (*P* = 0.04, *P* = 0.02, and *P* = 0.009, resp.).

**Figure 5:**
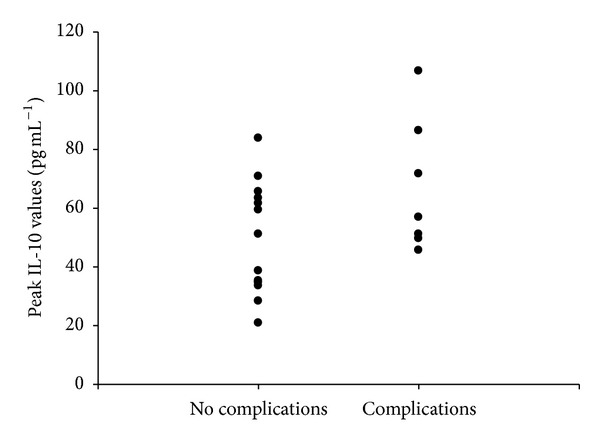
Scattergraph of peak postoperative IL-10 values in the seven patients who developed postoperative complications and in the 13 patients who did not. A trend for higher peak IL-10 values in the patients with complications was demonstrated (*P* = 0.09).

Outcome data of the 20 patients who participated in this secondary subgroup analysis are presented in Table 1. Overall RBC usage (units/patient) in the restrictive strategy group was 0 [0,2] (median [IQ range]) as compared to 1.5 [1,3] in the liberal strategy group (*P* = 0.037). Average postoperative hemoglobin concentration was 9.6 ± 1.1 g dL^−1^ in the restrictive group versus 10.7 ± 1.0 g dL^−1^ in the liberal group (*P* = 0.004). The duration of storage of transfused blood (in days) was shorter in the restrictive strategy group than in the liberal strategy group (21.7 ± 10.9 versus 28.5 ± 6.3, *P* = 0.044). The two groups did not differ in the time of mobilization, time of first liquid, and solid food intake as well as in the length of hospital stay (*P* = 0.414, 0.550, 0.139, and 0.643, resp.). Similarly, there was no difference in the rate of infectious complications between the two transfusion allocation groups. Finally, there was a trend for higher peak values of IL-10 in the seven patients who developed postoperative complications, although not statistically significant (*P* = 0.09) (Figure 5).

## 4. Discussion

The main finding of this secondary post hoc analysis was the higher level of IL-10 24 hours postoperatively in the group that received more blood transfusions intraoperatively and postoperatively in comparison to the restrictive transfusion group. Additionally, peak postoperative IL-10 levels were found to correlate with the units of blood transfused as well as the mean duration of storage and the storage time of the oldest unit transfused. In both transfusion allocation groups, there was a postoperative increase in the concentration of IL-6 and IL-10 in comparison to baseline.

RBC transfusion can be life-saving in severe hemorrhage, following major trauma or as a complication of major surgery and its benefits in these indications are undisputed. However, allogeneic blood products are a scarce and increasingly expensive resource, which is not risk-free. Among other risks, allogeneic blood transfusion has been incriminated in transfusion-associated immunomodulation, with initiation of a secondary inflammatory response enhancing the inflammatory insult evoked by the surgical procedure. The postoperative increase in the concentration of inflammatory cytokines demonstrated in our secondary analysis is in accordance with other studies which have shown ample release of various inflammatory mediators after surgery [9, 19]. In fact, it has been shown that the surgical trauma induces a profound inflammatory response through activation of complex cascade systems among which cytokines seem to play an important role in the acute phase. The release of these mediators is considered protective at least initially, since it aims at promoting healing of damaged tissues. However, the exaggerated and prolonged postoperative cytokine responses as well as any imbalance between proinflammatory and counterregulatory influences may lead to damage of otherwise healthy tissues and lead to the development of multiorgan failure and increased mortality [9, 20]. TNF-*α* is one of the first bioactive substances released and although it is not always detectable in the early phase following trauma probably due to its short half-life [9], it mediates the release of another proinflammatory substance, IL-6 [21–23]. IL-6 is released in response to a variety of stimuli, including major surgery and thermal injury [24]. It is a reliable marker of tissue injury, it is almost constantly detected postoperatively, and its systemic levels reflect the severity of the surgical impact [25–27].

It is not always easy to decide whether the postoperative cytokine surge is causally related to the extent of blood transfusion or to the circumstances that preceded or necessitated it. Thus, distinguishing the immunomodulatory effects of surgery from the effects of transfusion can be quite difficult. In our study, however, IL-6 showed similar plasma concentrations at equivalent time points postoperatively. The lack of differentiation between the two groups might imply that the surgical impact itself is predominantly responsible for IL-6 release and that the role of blood transfusion may be less definitive for IL-6 fluctuations postoperatively [9, 19, 28]. In contrast, although the initial pattern of IL-10 release was similar in both patient groups, there was a clear differentiation 24 h postoperatively in IL-10 levels between the two groups. By that time, IL-10 levels were significantly elevated in patients with excessive red blood cell supply. The observed difference in the postoperative time course and magnitude of IL-10 release may be largely attributable to the different transfusion therapy* per se.*

Although perioperative blood transfusion is thought to synergistically exaggerate the surgery-evoked cytokine response, it seems to induce a higher immunosuppressant than a proinflammatory effect. In clinical investigations, significant immunosuppression as a result of allogeneic blood transfusion has been suggested to contribute to the high recurrence rate of malignancies and to transplant rejection episodes [29]. The balance between proinflammatory and inflammatory cytokines is crucial for the host immune response and any derangement can lead to host defense failure [30] or enhance susceptibility to infectious complications [10, 11]. In fact, in the original randomized study, there was a tendency for an increased rate of respiratory infectious complications in the liberal transfusion group, although not statistically significant [17]. This trend was not observed in the subgroup analysis, obviously due to the low number of patients that were allocated to cytokine sampling. However, the trend for an increased rate of respiratory complications in the liberal transfusion group, as described in the original study, is consistent with literature reporting a dose-response relationship between the number of units transfused and the risk for postoperative infection [7, 28]. Both quantitative and qualitative immunologic alterations might predispose the recipient of a high blood transfusion volume to an increased risk for bacterial infections [7]. As already mentioned, blood transfusion has been shown to be associated with clinically important immunosuppression [10, 11], which may be mediated through the release or overexpression of IL-10. IL-10 is mainly considered anti-inflammatory and the predominance of anti-inflammation may lead to immunosuppression (“immunoparalysis”). IL-10 has been shown to downregulate a number of monocyte/macrophage actions and to prevent migration of polymorphonuclear leukocytes and eosinophils to sites of inflammation [15, 16, 31]. Additionally, high circulating levels of IL-10 impair leukocyte activation and degranulation [32]. IL-10 has also been suggested to play a role in downregulation and suppression of T-helper cell function [33, 34]. Immunosuppression mediated through IL-10 can increase mortality because it hampers the effective clearance of infectious agents in an experimental setting of bacterial pneumonia while inhibition of IL-10 bioactivity prolongs survival in a similar setting [35, 36]. Moreover, IL-10 predominance over proinflammatory mediators is correlated with poor patient survival after sepsis [37]. In our study, the possibility of a causal association between IL-10 and blood transfusion is further supported by the fact that, in this subanalysis, peak IL-10 values were found to correlate with the volume of transfused blood administered. The higher levels of IL-10, the time course of its release as well as in the greater incidence of postoperative respiratory complications in the liberal transfusion group in the original study, and the trend for higher peak values of IL-10 in the seven patients who developed postoperative complications in this subgroup analysis (although not statistically significant, probably due to the small number of patients sampled for cytokine measurements) might reflect the difference in transfusion policy between the two groups. Our results extrapolate data already shown in experimental studies to a clinical setting. Specifically, in an experimental study, allogeneic stored blood resulted in a significant TNF-*α* depression and IL-10 reduction when it was added to whole blood of a recipient and subjected to coculture, mimicking an* in vitro* model of blood transfusion [38]. Moreover, in a mice study, allogeneic blood transfusion led to a 5-fold increase in IL-10 production, which did not return to control levels before day 30 after transfusion [39]. Finally, Mynster presented* in vitro* evidence of reduced responsiveness of innate immune cells along with an increase in IL-10 production after incubation of freshly donated blood with allogeneic stored red blood cells [40].

In our subanalysis, peak IL-10 values were also found to correlate with the storage time of blood units administered. The generation of inflammatory mediators is, to some extent, affected by storage duration due to degeneration of leukocytes with increased storage time. With the disintegration of leukocytes, leukocyte-derived and other biologic response modifiers accumulate extracellularly during storage in a time-dependent manner and may play a significant role in immunosuppression and tissue damage [41, 42]. Erythrocytes also undergo many corpuscular changes during storage and the accumulation of toxic factors in the red cell membrane might also contribute to storage time-dependent dysregulation of immunity [43]. Moreover, in RBCs stored for a long time, depleted levels of 2,3 diphosphoglycerate with the resulting leftward shift in the oxyhemoglobin dissociation curve and altered RBC rheology may impede regional blood flow and adversely affect oxygen delivery to tissues [4, 44–46]. These may be involved in storage time-dependent infectious side-effects in the recipient by predisposing to splanchnic ischemia. Whether factors associated with duration of storage induce the observed increase in IL-10 release in the liberal transfusion group has yet to be established. To the best of our knowledge, it is the first time in the literature that such a correlation has been demonstrated. In fact, the tendency we found for an increased rate of respiratory complications (albeit without statistical significance) in the group with the greater volume of blood transfusion in the original study might alternatively be attributed to IL-10-storage-time- dependent dysregulation of immunity, since this group received blood of older age. This finding is in accordance with other observational studies relating duration of storage with morbidity [41, 47–50]. We cannot of course rule out the possibility that confounding might have affected our results since, in the design of our original protocol, the distribution of length of storage of blood units among the two groups was not truly random; according to our results, patients who were exposed to a higher volume of blood received a greater proportion of RBC units stored for longer periods as compared to patients who had fewer RBC transfusions. This however could be related to the fact that large transfusion requirements increase the possibility of transfusing blood units with long storage time. Additionally, our hospital blood bank tends to release the oldest RBC units first, following policies adopted by most hospital transfusion services. Thus, it is more likely for patients requiring a higher number or erythrocytes to receive transfusion with older units. However, we believe that the strength of the association between IL-10 values and storage variables in our study might imply a direct relation between IL-10 and age of blood administered. Additionally, multivariate regression analysis showed that both volume and age of blood transfused were independently associated with IL-10 values. A reliable way to eliminate the effect of any confounding and to detect a more solid association between storage duration of transfused blood and complications would be to design trials randomizing patients to different lengths of storage of transfused units. Such randomization however might be ethically unacceptable and therefore conclusions can mostly be reached from observational studies.

In contrast to IL-10 and IL-6, postoperative systemic concentrations of TNF*α* were only slightly elevated. This is consistent with the literature and may have to do with the sensitivity of the detection method involved (resulting in small differences in mediator levels to go undetected) or may be due to rises occurring only transiently during surgery; recovering by the time blood was sampled after surgery [9, 21]. Studies have demonstrated the postoperative induction of soluble TNF receptors, which may bind and inactivate TNF*α* [51]. IL-10 has also been shown to downregulate the production of TNF*α* from human alveolar macrophages and peripheral blood monocytes [52, 53]. In fact, in our study, the slight decrease in TNF*α* levels observed on the third postoperative day in the liberal transfusion group followed the surge of IL-10, which shows that the time course and variation of TNF*α* may be additionally regulated by the presence of anti-inflammatory IL-10.

The major limitation of this secondary post hoc analysis is that cytokines were analyzed in only a subgroup of patients due to the high cost of the measurement kits and to hospital budget limitations. We however believe that our results are relevant and give some insight especially into the potential association of IL-10 and transfusion-related parameters. Another consideration is that nonleukoreduced blood was used for transfusion, which could have had an impact on the levels of mediators studied. Despite the fact that the mechanisms involved in the immunomodulatory effect of allogeneic blood transfusion have not been thoroughly elucidated yet, it has been suggested that the majority of these effects is mediated by the interaction of white blood cells (or their products) in transfused blood and anti-leukocyte antibodies in the recipient plasma [54–56]. It has also been shown that patients transfused with blood without prestorage leukocyte reduction might present lymphocyte count alterations associated with a decrease in natural killer T-cells and therefore be at higher risk for postoperative bacterial infection episodes [57]. Therefore, TRIM seems to depend on the degree of contamination of transfused blood with leukocytes, storage time, and cytokine content [12, 38]. However, even though the reduction of leukocyte content in blood products by prestorage leukodepletion seems to be a reasonable approach to preventing TRIM, doubts have been raised against the universal implementation of leukocyte reduction. This is because immunomodulatory effects have been described even after transfusion of leukocyte-depleted blood [58, 59]. So, it has been argued that the remaining immunomodulatory effect of blood transfusion, even after prestorage leukodepletion, could be mediated either by the few remaining leukocytes or by unidentified bioactive substances other than those present in leukocytes. Such substances could be molecules in the plasma supernatant produced or released by platelet products and might mediated immune reactions on transfusion [60]. Additionally, erythrocytes might also play a role in immunosuppression, since erythrocyte membrane phospholipids have been shown to activate macrophage-derived phospholipids, which are potent immunoregulatory factors [4, 43, 45, 61, 62]. Moreover, allogeneic stored blood, with but also without leukodepletion, resulted in a significant TNF-*α* depression and IL-10 induction in an* in vitro* model of transfusion that used cultured human blood [38]. Therefore, the practice of universal leukocyte reduction has been questioned; it has not been adopted worldwide and it varies significantly among countries, taking into consideration economic factors as well [63–65]. Particularly in North America, a strong opposition against universal leukocyte reduction has been expressed by a large group of American blood bank physicians [66]. Prospective randomized studies could give some solid answers regarding the undoubtful benefit of leukodepletion, but these studies could be performed only in countries where the practice is not mandatory.

In conclusion, in the present subanalysis and taking into consideration the restrictions of the small sample size, a more liberal transfusion strategy was associated with higher IL-10 levels. Although it cannot definitely be stated that excessive transfusion therapy is responsible for the elevation of IL-10, the correlations observed show that the strength of the association between blood transfusion and IL-10 is greater than that for IL-6. Therefore, IL-10 with its potent anti-inflammatory effect may play a distinct role in the downregulation of host immunity and blood transfusion may exert its immunosuppressive effect in part by stimulating IL-10 production. However, a larger sample size and a more controlled analysis would be needed to address the question of transfusion-related immunomodulation and reproduce the findings of this preliminary report.

## Figures and Tables

**Table 1:** Outcome data in the 20 patients of the restrictive and liberal transfusion group who were sampled for perioperative cytokines.

Parameter	Restrictive strategy group (*n* = 10)	Liberal strategy group (*n* = 10)	*P* value
RBC usage (units/patient)	0 [0, 2]	1.5 [1, 3]	0.037
Average postoperative Hb (g dL^−1^)	9.6 ± 1.1	10.7 ± 1.0	0.004
Duration of blood storage (days)	21.7 ± 10.9	28.5 ± 6.3	0.044
Time of mobilization (days)	2 [1, 2]	1 [1, 3]	0.414
Time of first liquid intake (days)	2 [2, 3]	2.5 [2, 3]	0.550
Time of first solid intake (days)	3 [2, 4]	5 [3–5]	0.139
Length of hospital stay (days)	7 [5, 7]	7 [5, 10]	0.643
Pulmonary complications	1	4	0.303
Intra-abdominal collection	0	1	1.000
Urinary infection	0	0	1.000
Wound infection	0	1	1.000

Values are mean ± SD for parametric numeric data, median [25th–75th percentiles] for nonparametric numeric data, and number (percentage) for categorical data; RBC: red blood cells; Hb: hemoglobin.
